# Bisphenol A Activates Calcium Influx in Immortalized GnRH Neurons

**DOI:** 10.3390/ijms20092160

**Published:** 2019-05-01

**Authors:** Federico Alessandro Ruffinatti, Alessandra Gilardino, Valter Secchi, Erika Cottone, Davide Lovisolo, Patrizia Bovolin

**Affiliations:** 1 Department of Pharmaceutical Sciences, University of Eastern Piedmont, via Bovio 6, 28100 Novara, Italy; 2 Department of Life Sciences and Systems Biology, University of Torino, via Accademia Albertina 13, 10123 Torino, Italy; alessandra.gilardino@unito.it (A.G.); valter.secchi@studenti.unito.it (V.S.); erika.cottone@unito.it (E.C.); davide.lovisolo@unito.it (D.L.); patrizia.bovolin@unito.it (P.B.)

**Keywords:** bisphenol A, endocrine disruptors, calcium signaling, neuroendocrine cells, estradiol

## Abstract

Bisphenol A (BPA) is one of the most widely used chemicals worldwide, e.g., as a component of plastic containers for food and water. It is considered to exert an estrogenic effect, by mimicking estradiol (E2) action. Because of this widespread presence, it has attracted the interest and concern of researchers and regulators. Despite the vast amount of related literature, the potential adverse effects of environmentally significant doses of BPA are still object of controversy, and the mechanisms by which it can perturb endocrine functions, and particularly the neuroendocrine axis, are not adequately understood. One of the ways by which endocrine disruptors (EDCs) can exert their effects is the perturbation of calcium signaling mechanisms. In this study, we addressed the issue of the impact of BPA on the neuroendocrine system with an in vitro approach, using a consolidated model of immortalized Gonadotropin-Releasing Hormone (GnRH) expressing neurons, the GT1–7 cell line, focusing on the calcium signals activated by the endocrine disruptor. The investigation was limited to biologically relevant doses (nM–µM range). We found that BPA induced moderate increases in intracellular calcium concentration, comparable with those induced by nanomolar doses of E2, without affecting cell survival and with only a minor effect on proliferation.

## 1. Introduction

Among the many chemical molecules present in the environment that can interact with the endocrine system and have been classified as endocrine disruptors, Bisphenol A (BPA) is one of the most widely used, e.g., as a component of plastic containers for food and water. It is considered to exert an estrogenic effect, by mimicking estradiol (E2) action [1,2]. Low levels of BPA have been found in more than 90% of urine samples from human subjects in the US [3,4] and in about 50% of samples of milk from lactating women [5]. Because of this widespread presence, it has attracted the interest and concern of researchers and regulators, and a huge amount of studies has addressed its potential adverse effects. Despite this, the field is still open to controversy. There is a strong agreement on the levels of BPA that can be found in humans [2,3,5,6,7,8]: most reports converge on the range 0.3–10 ng/mL (i.e., nanomolar concentrations). On the other hand, BPA effects registered by the epidemiological studies are less univocal, in particular when dealing with potential neurological damages. Recently, a relationship between children exposure to several environmental toxicants, including BPA, and autism spectrum disorders has been proposed [9], but the evidence is limited. Several reviews dealing with data from humans have recently been published, some of them focusing specifically on effects on the nervous system [8,10,11,12,13]. Most of these reviews point to the limitations and inconsistencies of the available data, and on the difficulty to conciliate the limited information on humans with the findings from animal studies. As for the latter, and restricting to studies on effects on the nervous system, several groups have reported loss of synaptic contacts and plasticity, alterations in neuronal development and morphology in rodents [14,15,16,17,18] and fishes [19], and impairment of visual function in cats [20]. Of particular relevance, given the differences in metabolism and physiology of estrogens between rodents and primates, are the data reporting a loss of synaptic contacts in the monkey prefrontal cortex and hippocampus [21].

In vitro data referring to effects on neuronal models are even more scarce: a reduction in the differentiation of human stem cells into dopaminergic neurons has been reported [22]; and another study has reported effects on gene transcription in Gonadotropin-Releasing Hormone (GnRH) neurons and GT1–7 cells [23]. In the latter experimental model, BPA has been shown to induce oxidative stress [24].

The main bias that affects the animal and in vitro data is the extremely wide range of concentrations administered to animals or used in vitro. It has been shown that administration of 25 μg/kg to pregnant rats resulted in blood levels in the ng/mL range, i.e., the range reported in humans [6]. The current US-Environmental Protection Agency (EPA) reference dose (the daily dose that EPA calculates to be safe for humans over the lifetime) is 50 μg/kg/day [2]. However, adverse effects have been reported for lower doses, and, on the contrary, many of the data found in the literature are obtained at significantly higher doses [15,16,23,24], thus hindering their functional relevance. A further complication is due to the finding that the effects at low doses may be quite different, and even stronger, than those observed at higher concentrations [16,25]. As reported by a consensus panel [6]: “exposing tissues to only an extremely narrow range of doses of BPA may lead to erroneous conclusions … animal experiments on unstudied systems must avoid narrow dose ranges, especially the use of only a few very high doses”.

The field is therefore quite open, and while better designed studies on humans are quite unanimously considered of the utmost importance, the understanding and clarification of the mechanisms by which biologically realistic doses of BPA can exert potential interference with the endocrine and neuroendocrine systems is of high relevance. Interestingly, a large-scale cooperative project headed in the US by the NIEHS and FDA is currently operative [26], with the aim of collecting controlled and reproducible animal data using doses ranging from 2.5 to 25,000 µg/kg/day, i.e., exposures corresponding to blood levels from nM to µM concentrations.

Even if the hypothalamic-pituitary axis is the main controller of reproductive function, the data on the effects of BPA on GnRH neurons are quite limited. In addition to the two papers cited above [23,24], a few papers [27,28,29] point to indirect effects through kisspeptin neurons and consequent altered input on GnRH neurons; but here again the range of doses is quite wide. Another paper [30] reports no significant change in neuronal morphology and inputs at the US-EPA reference dose; it only exacerbated responses normally induced by E2. Finally, it has been found that, in rats, neonatal exposure to BPA affected ovarian development but not the ability of GnRH neurons to respond to steroid-positive feedback [31]. Therefore, to date, the data about the effects of low, environmentally relevant doses on GnRH neurons are far from complete.

One of the ways by which endocrine disruptors (EDCs) can exert their effects is the perturbation of calcium signaling mechanisms. Several calcium permeable channels have been reported to be affected by BPA exposure [32], both by changes in protein expression and by direct activation/inhibition mechanisms. Most data refer to non-neuronal models, and report different and in some cases conflicting effects, which apparently depend on the cellular model and doses used (up to hundreds of µM, see, e.g., [33]). For neuronal and neuroendocrine models, where the calcium signaling machinery is the key controller of hormone secretion, the data are quite limited, and again not univocal. Block of voltage-dependent calcium channels (VDCCs) in different cell types, including neurons, at EC_50_ values of 26–35 μM has been reported [34]. On the contrary, doses above 100 µM induced an increase in the intracellular calcium concentration, [Ca^2+^]_i_, in a hippocampus-derived cell line [35]. At more environmentally relevant doses (10–100 nM), BPA increased the calcium transients elicited by activation of NMDARs in a subpopulation (less than 10%) of primary hippocampal neurons [36]. In the pituitary cell line GH3, BPA in the nM range induces calcium oscillations comparable to those elicited by E2 [37]. Another group reported a reduction in spontaneous calcium oscillations in migrating GnRH neurons from nasal explants [38], but most effects were observed at a high dose (50 µg/mL).

We addressed the issue of the impact of BPA on the neuroendocrine system with an in vitro approach, using a consolidated model of immortalized GnRH neurons, the GT1–7 cell line, focusing our attention on the calcium signals activated by the endocrine disruptor and on the mechanisms involved, and limiting the investigation to biologically relevant doses (nM–µM range).

## 2. Results

### 2.1. Effects of BPA on Proliferation of GT1–7 Cells

Since it has been reported that BPA can alter cell cycle progression in endocrine cells [39], we tested the effects of a range of concentrations (from 10 nM to 5 µM) of the molecule on GT1–7 cells maintained in 10% FBS for 24 and 72 h.

**Figure 1:**
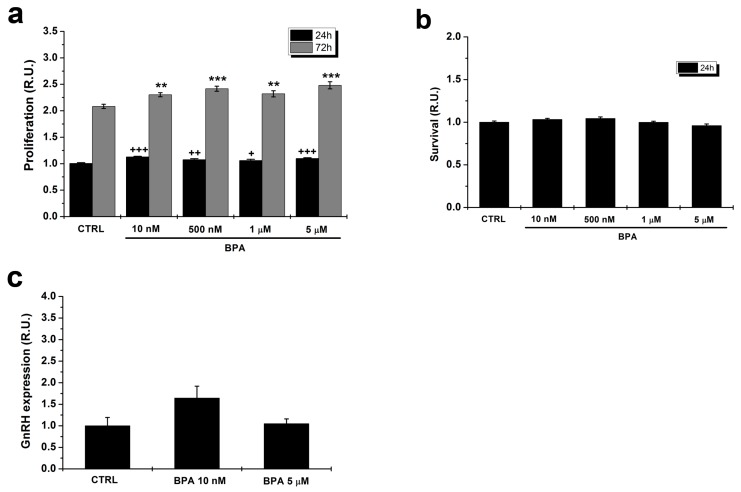
Effects of BPA concentrations in the nM–µM range on proliferation and survival of GT1–7 cells. (**a**) BPA induced a limited but statistically significant increase in cell proliferation in a concentration-independent way, at both 24 and 72 h. ANOVA and Dunnett’s post hoc test; * *p* < 0.05, ** *p* < 0.01, *** *p* < 0.001 for each BPA dose vs. control condition (CTRL) at 24 h; + *p* < 0.05, ++ *p* < 0.01, +++ *p* < 0.001 for each BPA dose vs. CTRL at 72 h. (**b**) The same concentration range had no effect on cell survival at 24 h (ANOVA and Dunnett’s post hoc test). (**c**) At both 10 nM and 5 µM, BPA did not significantly affect GnRH expression (*p* = 0.15; ANOVA and Dunnett’s post hoc test). R.U., Relative Units with respect to the control condition (CTRL).

Figure 1a shows that both at 24 and 72 h in culture BPA induced a significant but limited (about 10%) increase in the proliferative rate at all doses. Thus, the molecule only minimally affects cell cycle progression in the range usually considered to be relevant for animals and humans. Moreover, since the slight increase in number of cells could mask a combined positive effect on proliferative rate with a negative one on cell survival, we tested the effect of BPA on cells kept for 24 h in the presence of 0.5% FBS, a condition that promotes cell survival without significantly increasing cell proliferation. In these conditions, no significant difference in cell number could be observed between control and BPA-treated cells (Figure 1b).

### 2.2. BPA Does Not Affect GnRH Expression

Since GnRH expression is a key functional marker of GT1–7 cells, we investigated whether BPA affects this parameter by means of quantitative RT-PCR. As shown in Figure 1c, at both 10 nM and 5 µM the levels of gene expression were not significantly altered as compared with control cells.

### 2.3. BPA Induces Changes in [Ca^2+^]_i_

Next, we looked at the effects of BPA, in the same concentration range, on intracellular calcium homeostasis, a key parameter in the control of neuroendocrine cell function.

**Figure 2:**
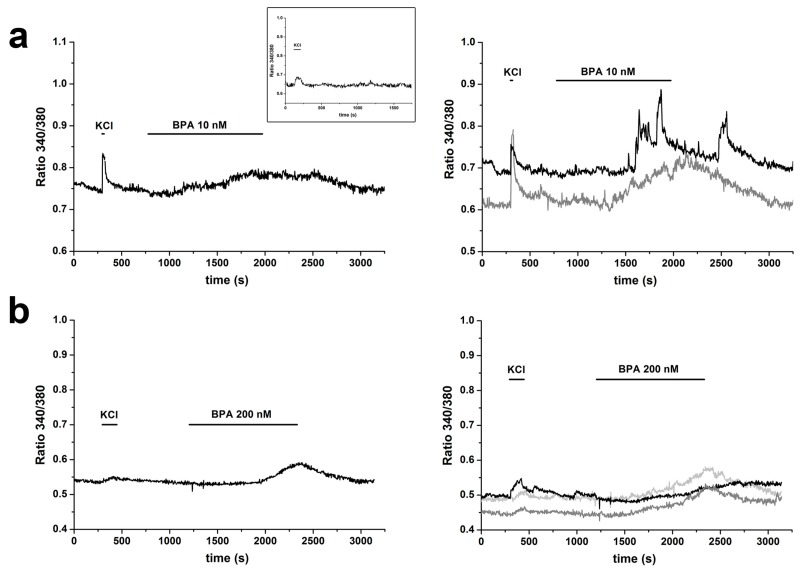

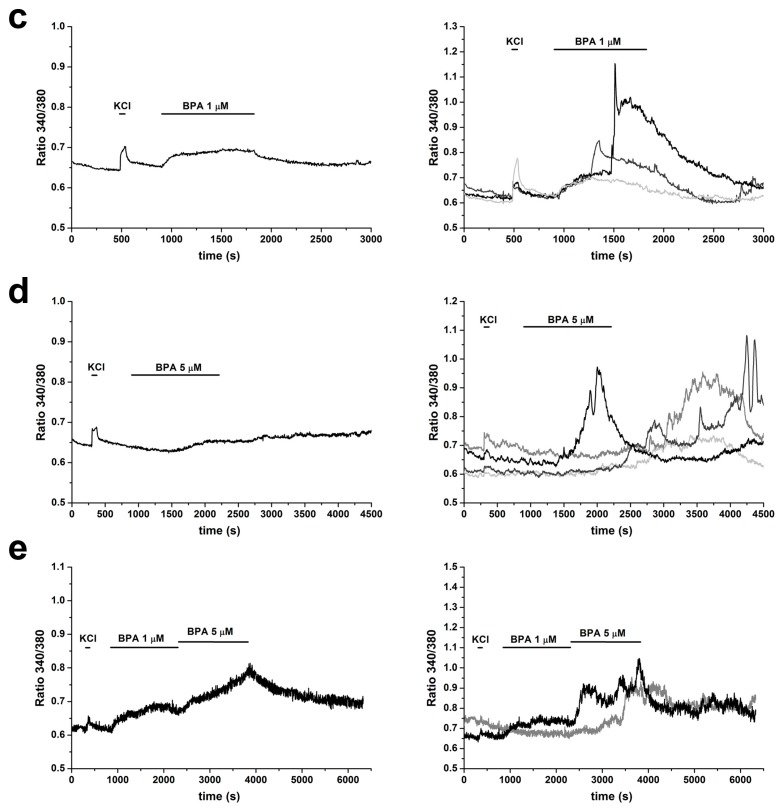
BPA-induced increases in cytosolic calcium concentration. (**a**–**d**) Changes in [Ca^2+^]_i_ in response to 10 nM, 200 nM, 1 µM and 5 µM BPA, respectively. (**Left**) Averaged responses from all cells from a single experiment. (**Right**) Examples of responses form single cells. Before the application of BPA, cells were chemically depolarized with 40 mM KCl and the increase in [Ca^2+^]_i_ due to influx through voltage-dependent channels was recorded. (**e**) Responses to incremental doses of BPA: (**Left**) averaged responses; and (**Right**) representative responses of individual cells. Inset in (**a**): averaged trace from *n* = 55 cells maintained for 30 min in normal physiological solution.

BPA induced dose-dependent changes in the intracellular calcium concentration, [Ca^2+^]_i_. In particular, four different BPA concentrations (10 nM, 200 nM, 1 µM and 5 µM) were tested in *m* independent experiments (*m* depending on the particular BPA dose, ranging from *m* = 6 to *m* = 11, for a total number of recorded cells ranging from *n* = 350 to *n* = 902). The left panel of Figure 2a–d shows examples of average responses at the four different doses; examples of responses from individual cells are shown on the right. In the presence of BPA, average responses were in most cases sustained and long lasting, showing a plateau; at the lower doses, the responses were partially reversible following washout of the agonist (Figure 2a–c). On the contrary, at 5 µM, the response was in most cases sustained even after washing (Figure 2d).

In four experiments, cells were challenged with incremental doses (1 and 5 µM); in two of them, the higher dose induced a further increase in [Ca^2+^]_i_ levels (Figure 2e). In this case, partial reversibility after washing was observed.

**Figure 3:**
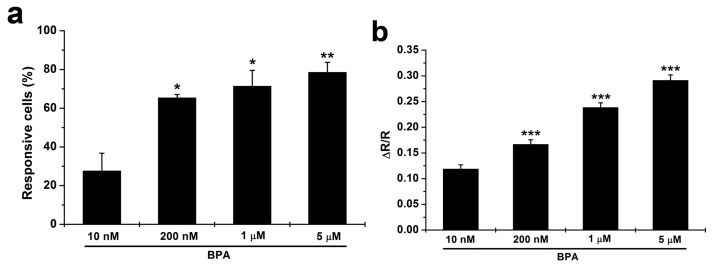
Both number of responding cells and amplitude of response were dependent on BPA concentration. (**a**) Percentage of responsive cells. Total cell number for each condition was *n* = 434 at 10 nM, *n* = 350 at 200 nM, *n* = 713 at 1 µM, *n* = 902 at 5 µM. Welch’s *F*-test and Games–Howell post hoc test; * *p* < 0.05, ** *p* < 0.01 for each BPA dose vs. the lowest one (10 nM). (**b**) Response peak amplitudes. Total number of responsive cells for each condition was *n* = 116 at 10 nM, *n* = 229 at 200 nM, *n* = 543 at 1 µM, *n* = 729 at 5 µM. Kruskal–Wallis *H*-test and Dunn’s post hoc test; *** *p* < 0.001 for all the possible pairwise comparisons.

The percentage of responding cells increased significantly and monotonically (from 27% to 79%) with BPA concentration (Figure 3a); the peak amplitude of the response also followed a similar dose-dependent trend, with ∆*R*/*R* ranging from 0.12 ± 0.01 to 0.29 ± 0.01 (Figure 3b).

A marked heterogeneity was present in the time course of the increases in [Ca^2+^]_i_: latencies and slope of rise of averaged responses showed a high degree of variability at all four concentrations tested. When analyzed at the single cell level, the heterogeneity was even more marked, even within the same experiment, as can be seen in the right panels of Figure 2a–d. In some cells, the responses started even after washout of the agonist.

In addition, the data presented on the right side of Figure 2 provide evidence that, at the single cell level, an oscillatory behavior could be observed in a limited percentage of the responsive cells. The percentage was not significantly different at all concentrations (mean values: 20%, 11%, 9%, and 10% at the four tested doses, respectively), even if in some experiments with stimulation by incremental doses the oscillatory behavior was present in some cells only at the higher dose tested (Figure 2e, right).

**Figure 4:**
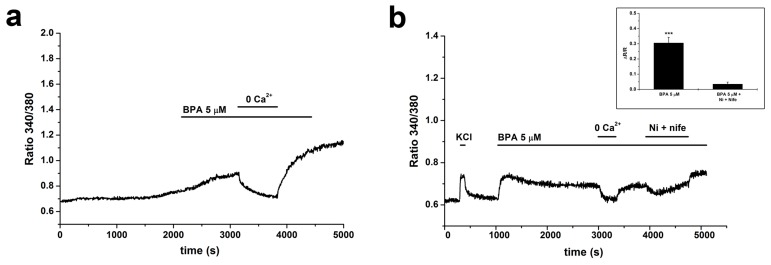
BPA-induced calcium signals are dependent on calcium influx, mainly through voltage-dependent channels. (**a**) Removal of calcium from the extracellular solution completely and reversibly abolished the [Ca^2+^]_i_ increase in response to 5 µM BPA. (**b**) Addition of 100 µM NiCl_2_ and 10 µM nifedipine strongly and reversibly reduced the [Ca^2+^]_i_ increase during perfusion with 5 µM BPA. Inset: Response amplitudes during BPA administration with and without addition of blockers of voltage-operated calcium channels. Wilcoxon signed-rank test for paired samples; *** *p* < 0.001.

The increases in [Ca^2+^]_i_ were totally dependent on influx from the extracellular medium, as shown in Figure 4a (one experiment representative of *m* = 4). To provide information on the channels involved in the generation of this influx, we performed three experiments (*n* = 140 cells; only cells that gave a response to KCl were analyzed) in which, during a sustained response to 5 µM BPA, 100 µM NiCl_2_ and 10 µM nifedipine, blockers, respectively, of T- and L-type VDCCs, were added to the medium. This protocol has been shown to block nearly all the calcium increase activated by chemical depolarization in these cells [40]. The combined blockers caused an average reduction of the [Ca^2+^]_i_ increase by 88.7% (Figure 4b).

### 2.4. Changes in [Ca^2+^]_i_ Induced by E2

To understand the potential role of the perturbation of calcium homeostasis induced by BPA in GT1–7 cells, it is necessary to compare the responses to BPA with those to the physiological estrogen, estradiol (E2). E2 has been considered to exert mainly an excitatory action on GnRH neurons [41,42,43,44] and in some cases even an inhibitory one, depending on cell activity [41]. Regarding the modulation of calcium signaling in a model of migrating GnRH neurons from nasal explants, both stimulation [45] and inhibition [38] of calcium transients have been reported.

**Figure 5:**
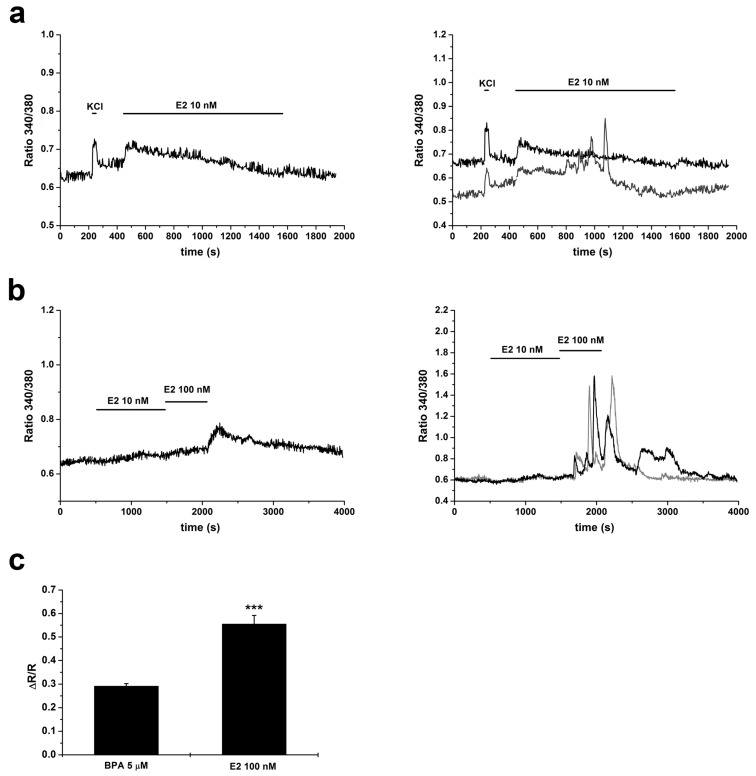
E2-induced increases in cytosolic calcium concentration. (**a**) Responses to 10 nM E2: (**left**) averaged responses from a single experiment; and (right) examples of responses of individual cells. (**b**) Cells were challenged to incremental doses of E2 (10 and 100 nM): (**Left**) averaged responses from a single experiment; and (**Right**) examples of responses of individual cells. (**c**) Comparison between the amplitudes of responses to 5 µM BPA (same data as in Figure 3b) and to 100 nM E2 (*n* = 181 cells). Mann–Whitney *U*-test; *** *p* < 0.001.

In GT1–7 cells, in *m* = 4 experiments 10 nM estradiol induced an increase in [Ca^2+^]_i_ in 72% of *n* = 279 cells; ∆R/R was 0.12 ± 0.01 (Figure 5a). When cells were subsequently stimulated with 100 nM E2 (Figure 5b), an additional response was observed in 94% of *n* = 181 cells; in this case ∆R/R was 0.56 ± 0.04, a value significantly higher than that obtained with the maximal dose of BPA (Figure 5c). Interestingly, while in 10 nM E2 the percentage of cells showing an oscillatory behavior was comparable to that observed with BPA (15%), with 100 nM E2 the percentage of cells showing marked oscillations in [Ca^2+^]_i_ was much higher, 77% (and the response lasted even after washout of the agonist).

## 3. Discussion

Environmental toxicants can affect the correct functioning of animal and human systems by interacting with different targets an interfering with a wide range of physiological processes. EDCs, in particular, are considered to exert their potentially adverse action by perturbing the endocrine system; among them BPA is one of the most widely investigated, based on its widespread use and its documented presence in human and animal fluids and tissues. The neuroendocrine axis is crucial for the correct growth, maturation and reproductive function in animals, but up to now has attracted only limited interest. The processes that can be affected at the cellular level cover a wide range, from differentiation and survival to electrical activity and hormone secretion; most of these are under the strict control of a critical parameter, the cytosolic calcium concentration, [Ca^2+^]_i_. Therefore, addressing this parameter can give relevant information about the pathways influenced by the molecule. Using a consolidated in vitro model on neuroendocrine cells, the GT1–7 cell line derived from mature mouse GnRH neurons [46], we have addressed the changes in [Ca^2+^]_i_ homeostasis induced by acute application of BPA. Due to the extremely wide range of concentrations that can be found in the literature, and to the fact that in many reports only one or two doses have been tested, we have chosen to use a relatively ample range of concentrations, in the interval considered to be compatible with BPA presence in animal and human organisms, based on epidemiological data, i.e., from nM to low µM concentrations.

We report that BPA, at all doses, does not affect cell survival and has a minor effect on cell proliferation. There are contrasting reports on BPA promoting cell proliferation [37] or apoptosis [35] in different experimental models, in many cases at high doses. In our case, the proliferative effect was statistically significant but of limited biological relevance. We also tested the potential effects of the molecule on GnRH expression, a key functional parameter in these cells. We found that at both the lower and higher dose employed in the present study, no significant changes in GnRH transcript levels could be detected; this finding is in accordance with data from other groups [23].

Most importantly, we found that the xenoestrogen induces changes in [Ca^2+^]_i_ that, particularly in the lower range, can be partially abolished following washing. Both the number of responding cells and the amplitude of response are dose dependent. In a subpopulation of cells, BPA induced an oscillatory response.

The response could be totally suppressed by switching to a calcium-free extracellular solution, indicating that the changes in [Ca^2+^]_i_ are totally dependent from influx from the extracellular medium.

Several families of ionic channels have been reported to be modulated, either positively or negatively, by BPA (for a recent and comprehensive review, see [32]). The data on calcium-permeable channels and neuronal cells are quite limited, and in some cases related to high BPA doses. GT1–7 cells are endowed with a rich array of calcium channels, both receptor- and voltage-operated; as for the latter, the tests performed in our experiments showing that chemical depolarization induces in most cells an increase in [Ca^2+^]_i_ provide evidence for a considerable expression of this family of channels. The finding that block of VDCCs abolished most of the response to BPA points to a major involvement of this family of channels in the changes in [Ca^2+^]_i_ induced by the xenoestrogen. VDCCs may be the direct target of the transduction pathways triggered by BPA, or open following the activation of other classes of depolarizing cationic channels.

Only two papers are available on BPA and neuronal voltage-dependent calcium channels, reporting conflicting results: one reporting an inhibitory effect in mouse DRG neurons [34], and another one about stimulation of neurotransmitter release dependent on VDCCs in PC12 cells [47]. It must be observed that both studies used very high doses, such as 100 µM and above.

It has been reported [38] that BPA reduces the spontaneous calcium spiking activity in GnRH neurons migrating from mouse nasal explants, and compared this effect with that of 10 nM E2, which they found also to be inhibitory. These data, obtained from a primary model of developing GnRH neurons, deserve a detailed comment. First, the Authors used neurons that showed spontaneous calcium signals. In our hands, GT1–7 cells only occasionally showed calcium spikes in basal conditions. It must be noted that, according to [41], E2 increases activity of primary GnRH neurons that are basally silent, while has an inhibitory effect on basally active neurons. Moreover, an excitatory effect of E2 (2–200 nM) has been described [45] in the same preparation as used in [38]. Finally, and of greater relevance in the present context, most of the data in [38] have been obtained from neurons stimulated with 50 µM BPA. Again, this is a quite high dose, about two orders of magnitude above the lowest observed adverse effect level (LOAEL) that, for in vitro experiments, has been calculated to be about 0.2 µM [48]. Therefore, the relevance of these findings may be dependent on the experimental model and, more significantly, may be limited by the high dose employed.

Since no data are available on the effects on calcium homeostasis exerted by E2 in GT1–7 cells, we compared the responses to BPA with those induced by the physiological estrogen. We report that E2, in the nanomolar range (10–100 nM), induced [Ca^2+^]_i_ increases comparable to those elicited by BPA, even if the amplitude with 100 nM E2 was significantly higher than with 5 µM BPA. Moreover, with 100 nM E2, a marked increase in the oscillatory behavior and in the duration of the responses was observed.

In conclusion, we provide the first evidence that, in a consolidated model of neuroendocrine cells, environmentally relevant doses of BPA can induce perturbation of intracellular calcium homeostasis; these changes are of moderate amplitude and, except for the higher dose, reversible after washing. Over about three orders of magnitude, the increase in peak amplitude of the responses was about twofold, and well below the amplitude of the signals induced by 100 nM E2. It should be noted that even subtle changes in calcium homeostasis can have significant functional consequences, such as changing the levels of cellular excitability and activation of intracellular enzymes. However, further investigations are needed to elucidate the differences in the signaling pathways and the biological outcomes implied by the different responses induced by BPA and E2 in terms of both signal amplitude and oscillatory behavior.

## 4. Materials and Methods

### 4.1. Materials

Unless otherwise specified, materials were obtained from Sigma-Aldrich (St. Louis, MO, USA).

### 4.2. Cell Culture

GT1–7 cells, an immortalized line derived from highly differentiated mouse GnRH neurons (generously donated by Prof. P.L. Mellon, Department of Obstetrics, Gynecology, and Reproductive Sciences, Center for Reproductive Science and Medicine, University of California San Diego, La Jolla, CA, USA), were maintained in Dulbecco’s Modified Eagle’s Medium (DMEM) supplemented with 10% heat-inactivated fetal bovine serum (FBS, Lonza, Basel, Switzerland), gentamycin (50 µg/mL), and glutamine (2 mM) at 37 °C, in a humidified atmosphere of 5% CO_2_ in air. BPA and E2 were dissolved in DMSO prior to dilution in media.

### 4.3. Survival and Proliferation Assays

For proliferation assays, GT1–7 neuronal cells were plated in 96-well plates in DMEM containing 10% FBS at a density of 40,000 cells/cm^2^ (Falcon, Becton Dickinson, Franklin Lakes, NJ, USA) and maintained in DMEM with 10% FBS. After 24 h, the medium was replaced with DMEM plus 10% FBS with or without different concentrations of BPA for further 24 or 72 h.

For survival assays, after one day in 10% FBS, the medium was switched to one containing 0.5% FBS, to promote survival without interference with proliferative effects; different concentrations of BPA were added for 24 h.

After the treatments, cells were fixed in 2.5% glutaraldehyde in PBS, stained with crystal violet (0.1% in 20% methanol), solubilized in acetic acid (10%, *v*/*v*) and read at 595 nm in a Microplate Reader (model 550, Bio-Rad Laboratories, Hercules, CA, USA).

Data (total cell number normalized to control) were expressed as mean ± standard error of the mean (SEM). Three independent experiments (each performed in 6 technical replicates) for each condition were carried out.

### 4.4. GnRH Expression Analysis

GT1–7 neuronal cells were plated in 60 mm tissue culture dishes (Falcon, Becton Dickinson, Franklin Lakes, NJ, USA) in DMEM containing 10% FBS at a density of 40,000 cells/cm^2^. After 24 h, the medium was replaced with DMEM plus 10% FBS with or without BPA 10 nM or 5 µM for further 72 h; three independent replicates were set for each condition. Total RNA was isolated from control and treated GT1–7 cells using Tri-Reagent (Sigma, St. Louis, MO, USA) and following manufacturer guidelines. qReal-Time PCR was performed using SensiFAST SYBR No-ROX kit (Bioline, London, UK) and the thermal cycler Rotor Gene Q (Qiagen, Hilden, Germany). Primers were designed with Primer-BLAST software (NCBI, National Center for Biotechnology Information, U.S. National Library of Medicine, Bethesda, MD, USA) and were as follows: GnRH Sense 5′-TCTTGATGTCCCTTAGAGTGG-3′, Reverse 5′-GCCCATCTCTTGGAAAGACT-3′; β-actin Sense 5′-TCTTTGCAGCTCCTTCGTTG-3′, Reverse 5′-ACGATGGAGGGGAATACAGC-3′. Each sample was analyzed in three technical replicates containing 50 ng of total RNA. The relative quantification of gene expression was done using a standard curve that was built by pooling all the RNA samples and making serial dilutions (range: 200–6.25 ng of total RNA). The amplicon concentrations were expressed in arbitrary units and were normalized for the expression of β-actin, a commonly used housekeeping gene. GnRH mRNA expression of BPA-treated cells was reported as average fold changes relative to the expression of control (DMSO treated) cells; controls were assigned a value of 1.

### 4.5. Calcium Imaging

For ratiometric measurements of [Ca^2+^]_i_ GT1–7 cells were plated on glass cover-slips (32 mm diameter) coated with poly-l-lysine (100 µg/mL) at densities of 10,000 cells/cm^2^. The cells were maintained in DMEM supplemented with 10% FBS, gentamycin (50 µg/mL), and 2 mM glutamine at 37 °C, in a humidified atmosphere of 5% CO_2_ in air and then switched for 4–5 days to 0.5% FBS supplemented with B27 (Invitrogen, Carlsbad, CA, USA), to improve survival and differentiation. Calcium imaging technique, protocols and instrumentation were as previously described [49]. Briefly, cells were loaded with the Fura-2 acetoxymethyl ester (2.5 µM, 45 min, 37 °C) and subsequently shifted to a standard physiological Tyrode solution of the following composition (in mM): NaCl, 154; KCl, 4; CaCl_2_, 2; MgCl_2_, 1; 4-(2-hydroxyethyl)-1-piperazineethanesulfonic acid (HEPES), 5; glucose, 5.5; and NaOH (pH 7.35). Agonists of interest (i.e., BPA and E2) were dissolved in the Tyrode solution at the required concentrations. Resulting solutions were applied with a microperfusion system; for calcium-free conditions, the CaCl_2_ salt was omitted and the calcium chelator ethylene glycol tetraacetic acid (EGTA; 0.5 mM) was added. Cells were imaged every 3 s at 37 °C using a monochromator system attached to a Nikon inverted microscope with a 20× objective (Nikon S Fluor). Images were acquired using an enhanced CCD camera (Roper Scientific/Photometrics, Martinsried, Germany) and the Metafluor software (Universal Imaging Co., West Chester, PA, USA). Calcium signals in response to the different stimuli were measured as peak amplitude in terms of increment in the ratiometric fluorescent emission (∆*R*), relative to its basal value (*R*).

Before application of either BPA or E2, cells were challenged with a Tyrode solution containing 40 mM KCl, to depolarize cells and elicit calcium influx trough voltage-dependent channels, as a test of cell viability.

### 4.6. Statistical Analysis

Mean and standard error of the mean (SEM) were used as measures of central tendency and dispersion, respectively. All samples were first tested for normality (Shapiro–Wilk test) and for homogeneity of variance (Levene’s test). When possible, statistical significance was assessed by parametric tests (*t*-test or one-way ANOVA and Dunnett’s post hoc test), otherwise non-parametric alternatives were used, as detailed in the text. Unless otherwise specified, all statistical tests were among unpaired samples, two-tailed and a *p*-value < 0.05 was considered statistically significant.
